# Barley domestication: the end of a central dogma?

**DOI:** 10.1186/s13059-015-0743-9

**Published:** 2015-08-26

**Authors:** Robin G. Allaby

**Affiliations:** School of Life Sciences, University of Warwick, Coventry, CV4 7AL UK

## Abstract

Genomic analysis of barley paints a picture of diffuse origins of this crop, with different regional wild populations contributing putative adaptive variations.

## Introduction

Barley did not come from any one place. Contrary to the common perception that domesticates originate from a single center associated with a single culture, a recent study published in *Genome Biology* by Poets et al. [1] shows that barley comes from a wide region spanning across the Levant and beyond. This finding has ramifications for our understanding of the evolution of domesticated crops.

There is a long history of domesticated crops being considered centric in their origins. At the time that Darwin was amassing evidence for the mechanism of evolution by natural selection, the common perception amongst farmers and breeders was that different varieties of domesticated organisms represented different domestication events from different species or wild variants. Darwin overthrew this notion, most famously with his demonstration that crossing different domesticated varieties of pigeons could reproduce a single wild form: the rock pigeon. His purpose was to prove that each of the domesticated varieties had been modified from a single wild ancestor as a pure lineage on which artificial selection had acted, thereby illustrating the power of small increments in the selection process.

Arguably, it was Darwin who originally invoked a centralized origin for most of our domesticated plants and animals, linking the domestication of each species back to a single ancestor and event. Although he had no notion of the mechanism of inheritance, his argument was essentially a genetic one. The concept of a single origin developed a geographical aspect with Vavilov’s Centers of Origin hypothesis. This hypothesis proposed that regional centers were associated with a number of domesticated forms arising together, so that centers of diversity were correlated with centers of origin. This concept was further developed in the context of single species, such that the origin of any one species was proposed to be associated with the area of highest genetic diversity within that species.

Subsequently, Childe added a cultural dimension, also with a centralizing narrative, in the form of the concept of a Neolithic Revolution. According to this concept, the rise of domesticated forms was associated with an elite group of proto-farmers. This would require that all of the crops within a ‘Neolithic Package’, the suite of half a dozen or so crops associated with the rise of agriculture, arose from a geographically restricted region over a short period of time, which fits in with Vavilov’s Centers of Origin hypothesis. The overlapping biogeographic distributions of the crops of the Near East were subsequently used to identify this core region as the northern reaches of the Fertile Crescent. Genetic evidence for a single origin for many crops was found in the mutations that cause domestication syndrome traits, or the features that define a plant as domesticated [2]. For example, the development of a tough rachis (the stalk that bears the grain) to prevent seed dispersal in cereals and the release of dormancy in pulses are both often under simple genetic control. In almost all cases the same mutation is found to be responsible for the syndrome trait in any one species, implying that a single domestication event was involved [2]. For many, such compelling evidence seemed to punctuate the end of the debate.

## Barley: the crop that would not fit

Poets and colleagues have recently demonstrated a pattern of genetic diversity in barley that runs entirely counter to the view of a centric origin [1]. Barley, they have shown, is the mosaic product of a number of wild populations that span across the Fertile Crescent and beyond. In fact, barley was always a problem crop for the Center of Origin view. In most crop species, all varieties share just one tough rachis mutation, providing the most compelling evidence for a single origin, but barley has two such mutations. Previously, this has been interpreted as a rare example of a polycentric origin [2]. Earlier work by the Morrell group [3] showed that the two tough rachis mutants in barley are associated with genetically distinct groups of domesticated barley, one associated with the West and the other with the East, suggesting that a core center area of origin probably did not apply to barley. Recent archaeological evidence has confirmed the existence of a hitherto unrecognized eastern area of the Fertile Crescent in the foothills of the Zagros Mountains where domestication occurred independently from that in other Levant regions [4].

## Centricity and genomics

A polycentric description of barley’s origin is still one of centricity, implying a specific locality of origin, or in this case two specific localities. Any one tough rachis mutant must come from somewhere specific; the attribution of the origin of the genome as a whole to the same locality, however, is a questionable inference that relies on the assumption of the sudden rise of domesticated forms along with strong selection for the mutant. Of course the same principle applies to crops in which only a single tough rachis mutant is known, such as wheat [2]. Archaeology has indicated that something more complex probably occurred: selection for a tough rachis was weak and slow in all the major cereals [5]. Furthermore, archaeology indicates that domesticated forms arose in different areas in parallel rather than in a core area alone [6]. These findings open up the possibility that, over the longer timescales involved of thousands rather than tens of years, different parts of any one crop genome may have come from different regions. The first genetic evidence that indicated this possibility came from a study of 18 genes in einkorn (*Triticum monococcum*), which showed that the domestication-associated alleles most probably originated from a wide range of areas in the northern ranges of the Fertile Crescent [7]. At a wider scale, using retro-element profiles, it was later shown that emmer wheat (*Triticum dicoccum*) appeared to be related to wild populations right across this geographical range [8].

Poets and colleagues have increased the resolution of the evidence further, examining 6152 single nucleotide polymorphisms (SNPs) in barley to give a truly genomic picture of the crop's origin. They divided wild barley into five regional metapopulations, corresponding to Mesopotamia, the Northern and Southern Levant, the Syrian Desert and Central Asia. They found that they can discern several regional groups of domesticated barley relating to Central Europe, Coastal Mediterranean, Asia and East Africa. Generally, the western (European) populations of domesticated barley are more related to western wild barley populations, and conversely the eastern (Asian) barley populations are more closely related to eastern wild barley populations. The big surprise is that all five of the wild metapopulations have contributed to all of the domesticated barley groups. The differences observed between the cultivated groups is in the degree of each wild population’s contribution. Poets and colleagues ruled out the most obvious confounding signal that could give rise to this overall picture — recent introgression from different wild groups into cultivated barley — by searching for linkage blocks that are shared between cultivated and wild populations. They found none.

The emergent picture of crop genomes that has come into focus has changed from one in which a center is identified by a single locus, to a polycentric view in which several genes are considered, to a profusion of loci with alleles heralding from different localities. It makes less sense to consider an origin to be polycentric when there are a large number of geographic areas involved that cannot, by definition, be considered as centric. Genomics is settling on a diffuse origin for domesticated barley, which may also reflect the truth for the origins of other crops, such as emmer and einkorn.

## The adaptive strength of a diffuse origin: regional specializations

Wild barley’s growing regions span a range of habitats, from grasslands of varying altitude through to desert regions. Each of these areas has its own locally adapted barley ecotypes. The geographic mosaic of the domesticated barley genome opens up the possibility that it may not just be neutral population processes and selection for specific domestication syndrome traits that give rise to the domesticated forms. Poets and colleagues examined the ancestral composition of the genomes of each of the regional domesticated groups and found striking patterns. For example, both Asian and Western barleys have a specific genome segment contributed by the wild barleys of the Syrian Desert. Similarly, the northern Mesopotamian wild barleys have contributed specific segments to Western and Asian barleys. The implication is that cultivated barley may have gained increased drought resistance or other qualities from such wild contributions. The resulting domesticated crop is genetically diverse, and quite possibly more robust in its ecogeographic range than it would have been if it had originated from just one locality. The next step will be to identify candidate genes that are associated with these genomic blocks to see if more can be understood about the qualities that were supplied to the crop by each region. A similar understanding of the origins of all the major cereals may have profound implications for food security.

## Concluding remarks

Is barley domestication the exception or the rule? The archaeological evidence and more limited genetic evidence from other crops suggests that it may well be typical. Data such as those supplied by Poets and colleagues are causing us to rethink how domestication evolved in crops. While there has been intense focus on domestication syndrome traits, this study shows that background diversity also had an important role to play in how the domesticated forms of crop species came about. The point is starkly made in rice, where contention continues on the origin of *Oryza indica*, in which all the domestication syndrome traits are derived from *Oryza japonica* but the genetic background is entirely Indian [9]. The level of genetic diversity found in barley implies that other processes occurred in addition to the unconscious selection of syndrome traits, which could reflect wider networks of human interaction than previously supposed. Is the idea of Centers of Origin over for crops? The prevailing evidence seems to be coming out in support of Harlan’s original description of ‘noncenters’ [10] for the cereals, but we have yet to understand how our ancestors produced such genetically diverse crops.
